# Acute effects of ambient air pollution on outpatient children with respiratory diseases in Shijiazhuang, China

**DOI:** 10.1186/s12890-018-0716-3

**Published:** 2018-09-06

**Authors:** Jie Song, Mengxue Lu, Liheng Zheng, Yue Liu, Pengwei Xu, Yuchun Li, Dongqun Xu, Weidong Wu

**Affiliations:** 1 School of Public Health, Xinxiang Medical University, Xinxiang, 453003 China; 2 Henan International Collaborative Laboratory for Health Effects and Intervention of Air Pollution, Xinxiang, 453003 China; 3 Xinxiang Medical University, Xinxiang, 453003 China; 4 Hebei Chest Hospital, Shijiazhuang, 050041 China; 5 National Institute of Environmental Health, Chinese Center for Disease Control and Prevention, Beijing, 100021 China

**Keywords:** Air pollution, Respiratory disease, Children, Outpatients, Time-series study

## Abstract

**Background:**

Associations between ambient air pollution and child health outcomes have been well documented in developed countries such as the United States; however, only a limited number of studies have been conducted in developing countries. This study aimed to explore the acute effects of five ambient air pollutants (inhalable particles [PM_10_], fine particles [PM_2.5_], sulfur dioxide [SO_2_], nitrogen dioxide [NO_2_] and 0zone [O_3_]) on children hospital outpatients with respiratory diseases in Shijiazhuang, China.

**Methods:**

Three years (2013–2015) of daily data, including cause-specific respiratory outpatient records and the concentrations of five air pollutants, were collected to examine the short-term association between air pollution and children’s respiratory diseases; using a quasi-Poisson regression generalized additive model. Stratified analyses by season and age were also performed.

**Results:**

From 2013 to 2015, a total of 551,678 hospital outpatient records for children with respiratory diseases were collected in Shijiazhuang, China. A 10 μg/m^3^ increase in a two-day average concentration (lag01) of NO_2_, PM_2.5_, and SO_2_ corresponded to an increase of 0.66% (95% confidence interval [CI]: 0.30–1.03%), 0.13% (95% CI: 0.02–0.24%), and 0.33% (95% CI: 0.10–0.56%) in daily hospital outpatient visits for children with respiratory diseases, respectively. The effects were stronger in the transition season (April, May, September and October) than in other seasons (the hot season [June to August] and the cool season [November to March]). Furthermore, results indicated a generally stronger association in older (7–14 years of age) than younger children (< 7 years of age).

**Conclusions:**

This research found a significant association between ambient NO_2_, PM_2.5_, and SO_2_ levels and hospital outpatient visits in child with respiratory diseases in Shijiazhuang, China.

## Background

Many epidemiological studies have reported that exposure to air pollution is associated with an increased risk for cardiovascular and respiratory diseases [1–5], even at concentrations less than the current health-based guidelines [6–8]. The Global Burden of Disease study identified air pollution as a leading cause of global disease burden, especially in developing countries [9, 10]. Lelieveld reported that ambient air pollution leads to more than 3 million premature deaths globally each year, and that China had the most premature deaths (1.36 million) [11].

As a result of rapid industrialization and urbanization in the past two decades, China is experiencing one of its worst air pollution situations. In the first quarter of 2013, China experienced extremely severe and persistent haze pollution, affecting an area > 1.3 million km^2^ and approximately 800 million individuals [12]. The annual average particulate matter < 2.5 μm in aerodynamic diameter (PM_2.5_) and particulate matter < 10 μm in aerodynamic diameter (PM_10_) concentrations were 141 μg/m^3^ and 303 μg/m^3^, respectively [13]. Shijiazhuang has been listed as the second-worst polluted city, with record-breaking daily average concentrations on January 12, 2013, of 771 μg/m^3^of fine particles (PM_2.5_) and 800 μg/m^3^ of inhalable particles (PM_10_). However, only a limited number of studies have investigated the health effects of such levels of air pollution.

It has been established that children are vulnerable to the effects of air pollution [14, 15]. Evidence suggests that ambient air pollution has the potential to increase the severity of respiratory diseases, particularly in children. Nhung [16] observed that all ambient air pollutants (PM_2.5_, PM_10_, PM_1_, SO_2_, NO_2_, NO_x_, O_3_, and CO) were positively associated with pneumonia hospitalizations in children. Statistically significant associations were observed for most pollutants, except for O_3_ and SO_2_. Moreover, stronger associations were observed in infants than in older children [16]. Another study found that four pollutants (PM_2.5_, PM_10_, NO_2_, and SO_2_) were significantly associated with hospital visits for acute upper and lower respiratory infections. A time-series analyses from Shanghai (China) found that an increase of 2.49 μg/m^3^ in black carbon was associated with a 7% (95% CI: 5–8%) increase in asthma admission [17]. Moreover, contrary to the study by Nhung, stronger associations were observed among older children [17]. Another study from China also found stronger associations in older children [18]. Despite mounting literature suggesting that air pollution may be associated with respiratory disease in children, information regarding the association remains limited. It is important, therefore, to determine the reasons for these inconsistent data, and to study the exact respiratory effects of air pollution on children, particularly in severely polluted cities.

In the present study, we conducted a time-series study to investigate the association between five ambient air pollutants (PM_2.5_, PM_10_, sulphur dioxide [SO_2_], nitrogen dioxide [NO_2_] and Ozone [O_3_]) and child respiratory outpatients in children in Shijiazhuang, China.

## Methods

Shijiazhuang, the capital of Hebei province, comprises eight urban and suburban districts, with a total area of 2206 km^2^ and a population of 4.55 million at the end of 2013. The study area was limited to the traditional four urban districts (469 km^2^). Approximately 2.19 million permanent residents include 0.31 million children (< 15 years of age) residing in these four districts in 2015.

### Hospital outpatient data

**Fig. 1:**
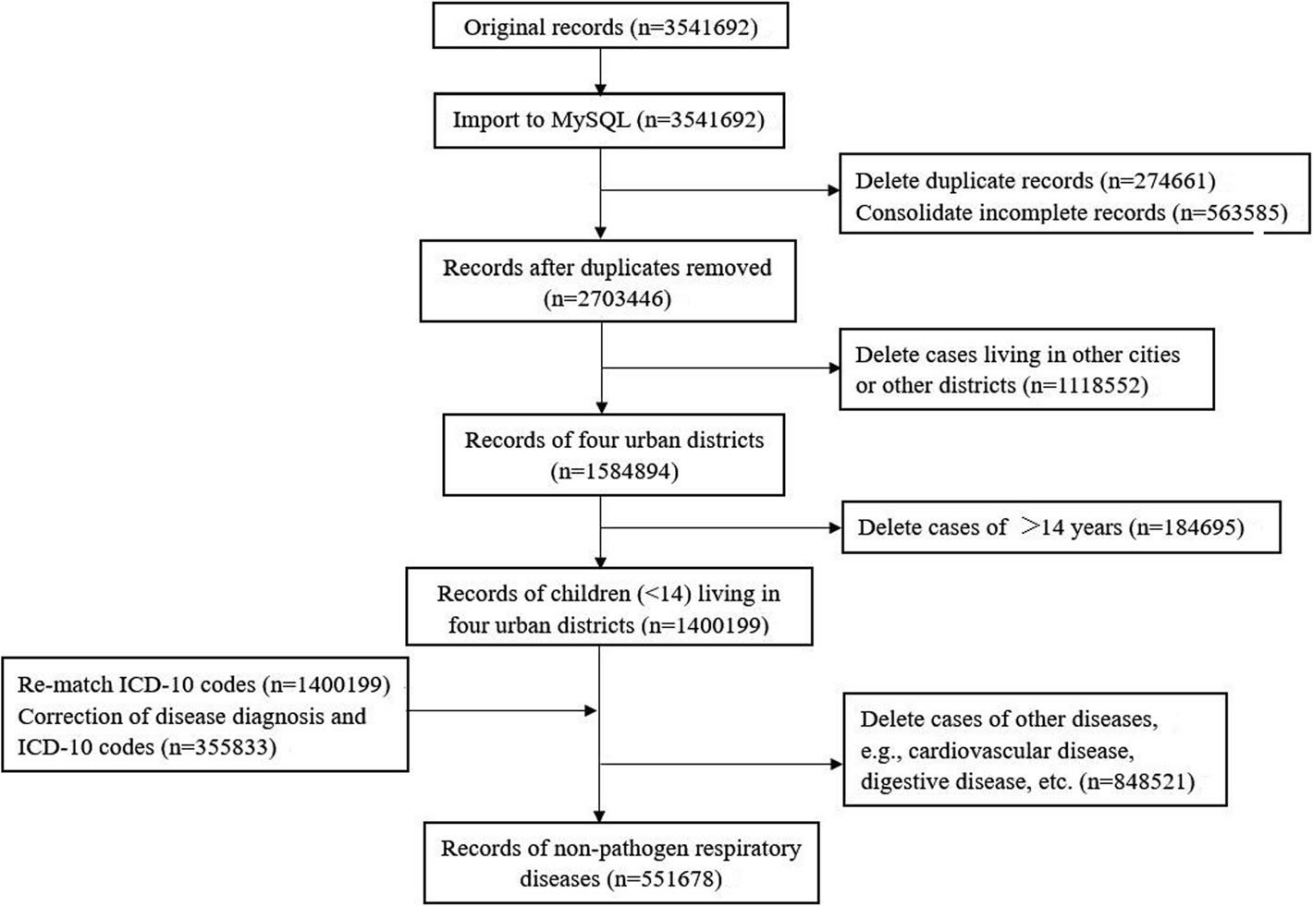
Data cleaning flow diagram

The Children’s Hospital of Hebei Province is the sole paediatric hospital in Shijiazhuang. Daily hospital outpatient visit data from January 1, 2013 to December 31, 2015 were collected from a database located at this hospital. All disease diagnoses were completed by computer coders. To validate health data, duplicate records were deleted and International Classification of Diseases, 10th Revision (ICD-10) codes were re-matched as reported in the authors’ previous research using MySQL server (version 5.6.26) [19]. The data cleaning strategy is described in Fig. 1. When there was a difference between the newly matched and original codes, this record would be picked up and discussed by a doctor’s team. Some errors, such as the wrong word, acronym or non-standard name, in the disease diagnosis would be changed to standard names and subsequently re-matched to an accurate ICD-10 code.

The respiratory outpatients’ data (ICD-10 codes J00-J99) were selected and targeted in the database. Patients residing outside of the four urban districts and those >14 years of age were excluded from the analysis. Outpatient visits caused by infection, suppuration, or ulceration were also excluded from this study. Finally, hospital respiratory outpatients’ visits (ICD-10 codes J00-J99, excluding pathogenic infections, abscess, suppuration, gangrenous and ulcerative diseases) and six specific or classified diseases (acute upper respiratory infections, ICD-10 codes J02-J06; pneumonia, J18; other acute lower respiratory infection, J20-J22; other diseases of upper respiratory tract, J30-J39; chronic lower respiratory diseases, J40-J47; and other respiratory diseases, J60-J99) were identified as health outcomes.

### Air pollution and meteorological data

Daily air pollution data, including PM_2.5_, PM_10_, SO_2_, NO_2_ and O_3_, were obtained from the website of China’s National Urban Air Quality Real Time Publishing Platform (http://106.37.208.233:20035/). The platform is administered by China’s Ministry of Environmental Protection. Hourly concentrations of each pollutant were measured from seven fixed site stations distributed in the four urban districts. These stations are mandated to be located away from major roads, industrial sources, buildings, and residential sources of emission from the burning of coal, oil or waste. This ensures that monitoring results reflect the urban air pollution level in the city rather than local sources of traffic or industrial combustion. The methods were based on the tapered element oscillating microbalance, ultraviolet fluorescence, chemiluminescence, ultraviolet fluorescence were used to measure PM (PM_2.5_ and PM_10_), SO_2_, NO_2_, and O_3_, respectively. For PM_2.5_, PM_10_, SO_2_, and NO_2_, daily concentrations were represented 24 h averages, and the O_3_ concentration was the maximal 8 h average from all valid monitoring sites in this study.

Daily mean temperature and humidity data were retrieved to adjust the effects of weather on hospital outpatients. Meteorological data were measured at a fixed site station and obtained from the Meteorological Bureau of Shijiazhuang.

### Statistical analysis

Time-series analysis is a regular analytic method to explore the acute effects of air pollution based on the daily aggregate date, and can control for both time-invariant and time-varying confounders by design [20].

The statistical analysis used a generalized additive model (GAM) to analyse the data. Because daily hospital visits typically followed an over-dispersed Poisson distribution, quasi-Poisson regression was used in the GAM [21]. Several covariates, including natural splines, were introduced to control for their potential confounding effects. First, a natural cubic regression smoothing function of calendar time with 7 degrees of freedom (*df*) per year excluded unmeasured long-term and seasonal trends longer than two months [20]. Second, a natural smooth functions of the mean temperature (6 *df*) and relative humidity (3 *df*) controlled for the nonlinear confounding effects of weather conditions [20]. Third, indicator variables were implemented for “day of the week” and public holidays. Briefly, the following log-linear GAM was fit to obtain the estimated pollution log-relative rate β in the selected city:

$$ logE\left({Y}_t\right)={\beta Z}_t+ DOW+ ns\left( time, df\right)+ ns\left( temperature, 6\right)+ ns\left( humidity, 3\right)+ intercept, $$

in which *E(Y*_*t*_*)* represents the expected number of respiratory disease outpatients at day *t*; *β* represents the log-related rate of respiratory diseases associated with a unit increase of air pollutants; *Z*_*t*_ represents the pollutant concentrations at day t; *DOW* is a dummy variable for day of the week; And *ns* indicates the natural cubic regression smooth function [22].

After establishing the basic model, single-pollutant models were initially used and introduced, a priori, in turn each air pollutant concentration on the concurrent day (lag0). To verify the stability of the model, three sensitivity analyses were conducted. First, alternative *df* were selected with 4–10 per year for the smoothness of time trends. Second, given that the health effects of ambient air pollutants could last for multiple days, more single lag days were used (lag1, lag2, lag3, lag4, lag5, lag6, and lag7) and moving average exposure of multiple days (lag01, lag02, lag03, lag04, lag05, lag06, and lag07). Third, two-pollutant models were built to examine the stability of the effect estimates after adjustment for co-pollutants. Co-pollutants with a correlation coefficient < 0.7 would be added to the two-pollutant model.

Both the total respiratory outpatients with non-pathogenic disease and cause-specific respiratory outpatients were assessed. Because behaviour patterns and common diseases may be different in children of different ages, all of these outpatients were stratified by age (0–3, 4–6, and 7–14 years). Because both air pollution levels and the incidence of respiratory disease events are known to vary by season, the analysis was stratified by cool season (November to March), hot season (June to August) and transition season (April, May, September and October), and reduced the *df* per year to 3, 2, and 3 respectively. The statistical significance of the differences between the effect estimates of the strata of a potential effect modifier (e.g., the difference between age or season) was tested by calculating the 95% confidence interval (CI) as$\left({\widehat{Q}}_1-{\widehat{Q}}_2\right)\pm 1.96\sqrt{{\left(\mathrm{S}{\widehat{\mathrm{E}}}_1\right)}^2+{\left(\mathrm{S}{\widehat{\mathrm{E}}}_2\right)}^2}$, in which ${\widehat{Q}}_1$ and ${\widehat{Q}}_2$ are the estimates for two categories, and $\mathrm{S}{\widehat{\mathrm{E}}}_1$ and $\mathrm{S}{\widehat{\mathrm{E}}}_2$ are their respective SEs [23]. Regardless of significance, modification of effect by a factor ≥ 2 was considered to be important and worthy of attention [23].

The statistical tests were two-sided, and effects with *p* < 0.05 were considered to be statistically significant. All statistical models were constructed using R software version 3.2.1 (R Foundation for Statistical Computing, Vienna, Austria) using the MGCV package. The effects are expressed as the percentage of change and 95% CI in daily hospital child respiratory outpatient visits per 10 μg/m^3^ increase in pollutant concentrations.

## Results

### Data description

A total of 3,541,692 total hospital outpatient records were retrieved for the period 2013 to 2015, from the Children’s Hospital of Hebei Province. A total of 1,400,199 records remained after deleting duplicate data, the records of cases residing outside of Shijiazhuang, and cases>14 years of age. After ICD-10 code re-matching, approximately 355,833 (25.4%) recodes were mismatched. Finally, 551,678 records of hospital outpatients caused by non-pathogen respiratory diseases were extracted. The percentages of total non-pathogen respiratory hospital outpatients according to age group were 72.2% for 0–3, 18.5% for 4–6 and 9.3% for 7–14 years of age, respectively. Acute upper respiratory infections (ICD-10 codes J00-J06) accounted for 37.4% of the total of non-pathogen respiratory diseases. Other acute lower respiratory infections (ICD-10 codes J20-J22) accounted for 36.7%, while pneumonia (ICD-10 codes J18) accounted for 12.1%. Other diseases of upper respiratory tract (ICD-10 codes J30-J39) accounted for 10.2%, chronic lower respiratory diseases (ICD-10 codes J40-J47) accounted for 3.0%, and other respiratory diseases (ICD-10 codes J60-J99) accounted for 0.7%.

During the study period, there were no missing value days for air pollutant measurements, meteorological variables, or health data. According to the results of the Shapiro-Wilk test, all of these data were skewed (i.e., non-normally distributed); therefore, median and quartile values were used to describe their distribution. Descriptive statistics from this study are summarized in Table 1. There was serious air pollution in Shijiazhuang, especially from PM_2.5_ and PM_10_, and on most days, these two pollutant concentrations exceeded the National Ambient Air Quality Standards (24 h average standards for PM_2.5_ is 75 μg/m^3^, PM_10_ is 150 μg/m^3^, SO_2_ is 150 μg/m^3^, NO_2_ is 80 μg/m^3^, and O_3-8h_ is 160 μg/m^3^). The highest daily average concentrations were 10.3 and 5.6 times the limit values, respectively, confirming that the main air pollutants in the selected city are, in fact, PM_2.5_ and PM_10_. The minimal, mean, and maximal daily average temperature and relative humidity were − 7.7 °C, 14.5 °C, 34.7 °C and 11.5%, 57%, 98%, respectively, reflecting the warm temperate continental monsoon climate in Shijiazhuang.

**Table 1:** Summary statistics of daily air pollutants, weather conditions, and children hospital outpatients caused by respiratory diseases (*N* = 551,678) in Shijiazhuang from 2013 to 2015

	Min	P25	P50	P75	Max
Air pollutant concentration (μg/m^3^)^a^					
NO_2_	13	36.8	51.9	71.6	176.8
O_3_	3.3	34.1	69.0	115.8	262.4
PM_10_	22.3	146.6	226.7	334.5	842.1
PM_2.5_	9.8	65.2	109.6	166.6	771.3
SO_2_	5.3	31.5	56.7	118.3	319.3
Meteorological measures					
Temperature (°C)	−7.7	5.0	16.0	24.2	34.7
Humidity (%)	11.5	43.0	58.4	72.3	98.0
No. of daily respiratory outpatients (J00-J98)^b^	243	389	442	527	915
Acute upper respiratory infections (J00-J06)	67	159	183	206	294
Pneumonia (J18)	7	33	45	71	180
Other acute lower respiratory infections (J20-J22)	68	124	149	216	400
Other diseases of upper respiratory tract (J30-J39)	6	35	47	59	150
Chronic lower respiratory diseases (J40-J47)	1	10	14	18	47
Other respiratory diseases (J60-J99)	0	2	3	5	13
Age (N)					
0–3	201	281	315	377	703
4–6	14	66	86	109	194
7–14	13	33	42	55	107
Season (N)					
hot (Jun to Aug)	285	366	407	437	551
Transition (Apr, May, Sep and Oct)	263	403	443	476	690
Cool (Nov to Mar)	243	408	562	700	915

^a^24-hour average for PM_2.5_, PM_10_, SO_2_, and NO_2_; maximal 8-h average for O_3_

^b^respiratory diseases except for pathogen infectious, abscess, suppuration, gangrenous and ulcerative diseases

**Fig. 2:**
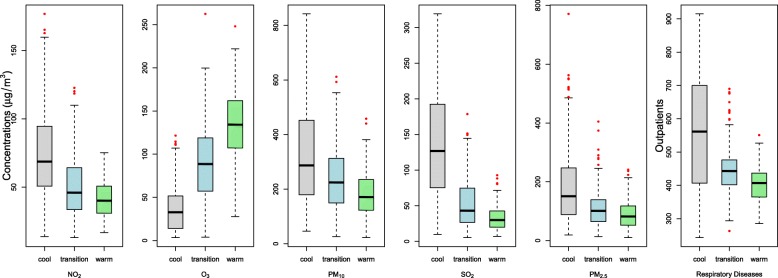
Box plots of five air pollutans in the cool, transition and warm season. Boxes indicate the interquartile range (25th percentile – 75th percentile); lines within boxes indicate medians; whiskers below boxes represent minimum values; whiskers and dots above boxes indicate maximum values

As shown in Fig. 2, daily air pollution concentrations (except for O_3_) and total respiratory outpatients were highest in the cool season and lowest in the hot season. The interquartile range of PM_2.5_, PM_10_, SO_2_ and NO_2_ concentrations in the cool season (158, 272.7, 117.1 and 43.8, respectively) were significantly higher than in the hot season (65.2, 110.8, 22.6 and 19.6, respectively).

Generally, there were strong correlations among PM_2.5_, PM_10_, SO_2_ and NO_2_ pollutants with the Spearman correlation coefficients, ranging from 0.50 to 0.75. PM_2.5_, PM_10_, SO_2_ and NO_2_ concentrations were negatively or weakly correlated with temperature and relative humidity. Maximal 8 h mean O_3_ concentrarion was negatively correlated with PM_2.5_, PM_10_, SO_2_ and NO_2_ (Spearman correlation coefficients ranged from − 0.31 to − 0.50), weakly correlated with relative humidity, and strongly correlated with temperature (*r* = 0.82, *p* < 0.05).

In the whole-season analysis, SO_2_, NO_2_ and PM_2.5_ were significantly associated with increased total respiratory outpatient visits. An increase of 10 μg/m^3^ in two-day average concentrations of SO_2_, NO_2_ and PM_2.5_ corresponded to a 0.33% (95% CI: 0.10–0.56), 0.66% (95% CI: 0.30–1.03), and 0.13% (95% CI: 0.02–0.24) increase in total respiratory outpatient visits (Table 2). The associations between O_3_, PM_10_ and total respiratory outpatients were positive but non-significant. For cause-specific diseases, positive associations were observed except for correlations between O_3_ and chronic lower respiratory diseases (ICD-10 codes J40-J47) and other respiratory diseases (ICD-10 codes J60-J99).

**Table 2:** Percent change (95% CI) in children hospital outpatients caused by total and cause-specific respiratory diseases per 10 μg/m^3^ increase in concentrations of five air pollutants in Shijiazhuang, China, 2013–2015

	Total	Acute upper respiratory infections (J00-J06)	Pneumonia (J18)	Other acute lower respiratory infections (J20-J22)	Other diseases of upper respiratory tract (J30-J39)	Chronic lower respiratory diseases (J40-J47)	Other respiratory diseases (J60-J99)
NO_2_	**0.66 (0.30,1.03)**	0.18(−0.3,0.67)	**0.78 (0.2,1.36)**	**0.57 (0.09,1.05)**	**2.25 (1.21,3.3)**	0.62(− 0.66,1.91)	1.66(− 0.97,4.3)
O_3_	0.20(− 0.12,0.51)	0.24(− 0.13,0.62)	0.08(− 0.53,0.68)	0.14(− 0.31,0.59)	0.72(− 0.16,1.59)	−0.4(−1.49,0.68)	−1.32(−3.35,0.7)
PM_10_	0.04(− 0.04,0.11)	0.01(− 0.08,0.11)	0.08(− 0.04,0.19)	0.04(− 0.05,0.13)	0.07(− 0.14,0.27)	0.07(− 0.18,0.31)	0.34(− 0.16,0.83)
PM_2.5_	**0.13 (0.02,0.24)**	0.12(− 0.02,0.27)	**0.19 (0.02,0.36)**	0.12(− 0.02,0.26)	0.11(− 0.2,0.42)	0.24(− 0.14,0.62)	0.51(− 0.23,1.24)
SO_2_	**0.33 (0.10,0.56)**	0.18(− 0.14,0.49)	0.34(− 0.02,0.7)	0.22(− 0.08,0.52)	**1.14 (0.5,1.79)**	0.11(− 0.7,0.92)	0.81(− 0.81,2.43)

Significant statistical estimates are highlighted in bold

### Effects by season

The effect estimates of ambient air pollution on total respiratory outpatients showed significant differences among three seasons. Effect estimates of all five pollutants were significant in the transition season, and non-significant in both the cool and hot seasons, except for SO_2_ in the hot season. NO_2_, PM_2.5_, PM_10_ and O_3_ exhibited highest effects in the transition season. The magnitudes of SO_2_-associated increase were approximately 2 times higher in the hot season than in the transition season. Significant differences were observed for SO_2_ and O_3_ between the cool season and the hot, transition season, for PM_10_ in the transition season and the cool and warm season, for NO_2_ in the cool season and the transition season.

### Effects by age

The percent increase in associations between air pollutants and total respiratory hospital outpatients varied by age group. For NO_2_, PM_2.5_ and PM_10_ pollutants, the older the child was, the higher the effect estimates. O_3_ had significant influence on children 4–6 years of age, then on those 7–14 years, and the smallest on those 0–3 years of age. There were no significant effects of air pollutants on children 0–3 years of age, except for SO_2_. Meanwhile, significant effects were observed in children 4–6 and 7–14 years of age, except for SO_2_ and O_3_, respectively. Three pollutants (NO_2_, PM_10_ and O_3_) present significant differences between the 0–3 years of age group and the 4–6/7–14 years group, while differences in the other two pollutants were non-significant among the three groups (Table 3).

**Table 3:** Percent change (95% CI) in children hospital outpatients caused by respiratory diseases per 10 μg/m3 increase in concentrations of five air pollutants stratified by season and age in Shijiazhuang, China, 2013–2015

	Season			Age		
	Cool season	Hot season	Transition season	0–3	4–6	7–14
NO_2_	0.17(− 0.44, 0.78)^b^	0.71(− 0.65, 2.06)	**1.47 (0.69, 2.25)** ^b^	0.22(− 0.14, 0.59)^d,e^	**1.59 (0.86, 2.31)** ^d^	**2.39 (1.38, 3.39)** ^e^
O_3_	− 3.47(−4.38, −2.55)^a,b^	0.26(− 0.16, 0.67)^a^	**0.54 (0, 1.07)** ^b^	0.05(− 0.27, 0.37)^d^	**0.78 (0.16, 1.40)** ^d^	0.11(− 0.75, 0.98)
PM_10_	0.02(− 0.09, 0.14)^b^	−0.08(− 0.36, 0.2)^c^	**0.24 (0.09, 0.39)** ^b,c^	−0.03(− 0.10, 0.04)^d,e^	**0.19 (0.05, 0.34)** ^d^	**0.27 (0.07, 0.47)** ^e^
PM_2.5_	0.16(−0.01, 0.33)	−0.1(− 0.54, 0.35)	**0.32 (0.02, 0.62)**	0.06(− 0.05, 0.17)	**0.30 (0.08, 0.53)**	**0.38 (0.07, 0.68)**
SO_2_	−0.05(− 0.39, 0.29)^a,b^	**2.09 (0.99, 3.2)** ^a^	**1.05 (0.42, 1.68)** ^b^	**0.26 (0.03, 0.49)**	0.38(−0.08, 0.84)	**0.83 (0.19, 1.47)**

We used current day temperature and humidity (lag0) and 2-day moving average of air pollutant concentrations (lag01). Significant statistical estimates are highlighted in bold

^a^The difference between cool season and hot season was significant at α = 0.05. ^b^The difference between cool season and transition season was significant at α = 0.05. ^c^The difference between hot season and transition season was significant at α = 0.05. ^d^The difference between 0 and 3 years of age and 4–6 years of age was significant at α = 0.05. ^e^The difference between 0 and 3 years of age and 7–14 years of age was significant at α = 0.05. f The difference between 4 and 6 years of age and 7–14 years of age was significant at α = 0.05

### Sensitivity results

**Fig. 3:**
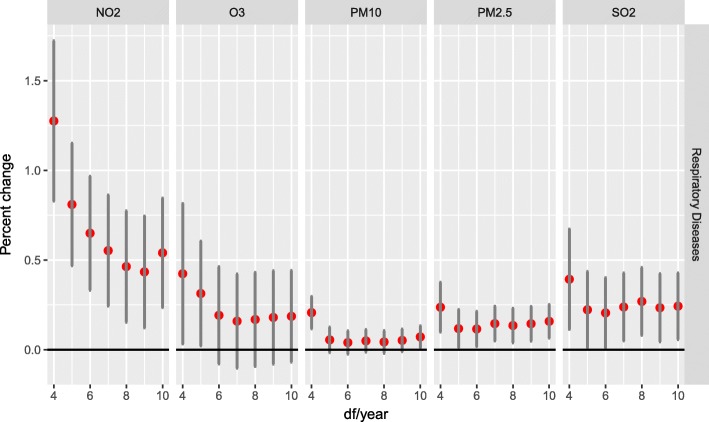
Percent increase of hospital outpatient visits with 10 μg/m^3^ increase of NO_2_, O_3_, PM_10_, PM_2.5_ and SO_2_ due to respiratory disease classified by degrees of freedom per year

The results of sensitivity analyses, adjusted for different *df* are shown in Fig. 3. The effect estimates remained stable. The results demonstrated that the acute effects of air pollution did not change substantially with the adjustment of smoothness of time using alternative *df* from 4 to 10 per year.

**Fig. 4:**
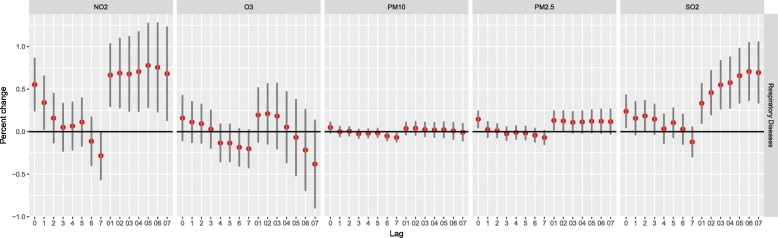
Percent increase of hospital outpatient visits with 10 μg/m^3^ increase of NO_2_, O_3_, PM_10_, PM_2.5_ and SO_2_ due to respiratory disease in different lag days

The results from the single-lag day (lag0-lag7) and cumulative exposure models (lag01-lag07) for the percent increase in children respiratory outpatients per 10 μg/m^3^ increase in pollutants are shown in Fig. 4. Statistically significant results were observed at lag 0, 1 and 01–07 day for NO_2_. Lag 0 and 01 day for PM_2.5_. Lag 0 and 01–07 day for SO_2_. respectively. For all five pollutants, the effects on cumulative days were higher than single-lag days. According to previous studies, lag0 day or lag01 air pollution was most closely correlated with child hospital outpatient visits. Therefore, a two-day average (lag01) exposure model was used for modifying effects analyses.

The results of the two-pollutant models using exposure at lag 01 are provided in Table 4. The magnitudes of all five pollutants were stable. Effect estimates of NO_2_, SO_2_ and PM_2.5_ pollutants remained statistically significant when adjusting for co-pollutants.

**Table 4:** Percent change (mean and 95% confidence intervals) of daily total respiratory outpatients associated with10ug/m^3^ increase of pollutant concentrations in single and two-pollutant models

Pollutants	Two-pollutant models	Estimates
NO_2_	Without adjustment	0.66 (0.30,1.03)**
	Adjusted for O_3_	0.73 (0.35, 1.11)**
O_3_	Without adjustment	0.20(−0.12, 0.51)
	Adjusted for SO_2_	0.29(−0.03, 0.61)
	Adjusted for PM_2.5_	0.24(−0.08, 0.56)
	Adjusted for PM_10_	0.21(−0.11, 0.53)
	Adjusted for NO_2_	0.31(−0.01, 0.63)
PM_10_	Without adjustment	0.04(−0.04, 0.11)
	Adjusted for O_3_	0.04(−0.03, 0.11)
PM_2.5_	Without adjustment	0.13 (0.02, 0.24)*
	Adjusted for O_3_	0.14 (0.03, 0.25)*
SO_2_	Without adjustment	0.33 (0.10, 0.56)*
	Adjusted for O_3_	0.37 (0.14,0.61)*
	Adjusted for PM_2.5_	0.26 (0.01, 0.53)*

Two-day moving average (lag01) concentrations of pollutants were used. *p < 0.05, **p < 0.001

## Discussion

Although the associations between ambient air pollution and daily hospital child outpatient visits have been well described in developed countries, studies in developing countries, especially in severely pollution haze Chinese cities, remain limited. The present study demonstrated that season and age may modify the health effects of air pollution in Shijiazhuang. Unlike other study results, the association between air pollution and daily children outpatient visits was generally more evident in the transition season than the hot or cool seasons.

The effect estimates of our results are lower than reported in previous studies [16, 24–26]. There are several potential reasons for this heterogeneity. First, the lower estimates may reflect the fact that Shijiazhuang’s air pollution was significantly more severe than in developed countries and other developing cities in China, which may reflect the shape of the concentration-response curves where there may be a flattening (saturation) at the higher end [27]. Second, the chemical components of PM pollution are very important to their effects on health, which may partially explain the reason for different effects among cities [21]. Third, the varying magnitude of misclassification of clinical diagnosis, as well as other factors such as statistical models and population characteristics, may explain the differences between our results and previous studies [28].

For the first time, the present study observed that the association between air pollution and daily child respiratory hospital outpatient visits in the transition season is significantly more sensitive than in hot or cool seasons. The concentrations of SO_2_, NO_2_, PM_2.5_ and PM_10_ were higher in the cool season, medium in the transition season, and lower in the hot season (Fig. 2). Associations between ambient air pollution and daily total non-pathogen respiratory outpatient visits were strongest during the transition season: the effect estimates were 2–6 times higher than in all seasons. The pattern of exposure to ambient air pollution in children may change from season to season [29]. Because of low temperatures, high air pollutant concentrations, and the use of central heating systems in the winter, residents generally stay indoors and close their windows. Similarly, high temperatures and the widespread use of air conditioning forces individuals to enter rooms and close windows. Thus, the exposure dose may be reduced in the cool or hot seasons. One study reported that the indoor /outdoor ratio of air pollutant concentration in Beijing (China) is 0.5 and 0.7 in the cool and hot seasons, respectively (data not shown). In contrast, the climate is more pleasant in the transition season; children’s outdoor activities and time with open windows in homes would be increased; therefore, exposure to ambient air pollution would likely be higher.

Previous studies have reported that the health effects of air pollution on infants and young children may be greater than in adults [30–32]. However, we found a very interesting phenomenon in our study: the effect estimate increases with age in children. Considering the differences in activity range and air pollution patterns among children of different age groups, our results may be easier to understand. Children 0–3 years of age need adult supervision and their activities are mainly indoor; consequently, their exposure to ambient air pollution is the least. Kindergarten (4–6 years) can offer a wide range of free activities, meanwhile the children are compliant and follow teachers’ recommendations to stay indoors when the air quality is inadequate. Children 7–14 years of age engage in the highest activities but have a weak awareness of self-protection, therefore, exposure dose may increase with age. Although the exact air pollution exposure dose to children of different ages remains unclear, this phenomenon provides new insights into research investigating the adverse health effects of air pollution on children, and warrants careful future study.

Another important finding from our study was that pollutant states may contribute to seasonal differences. Effect estimates of gaseous pollutants (SO_2_, NO_2_ and O_3_) with total respiratory outpatient visits in the hot season were higher than those in the cool season, while the PM pollutants (PM_2.5_ and PM_10_) had lower estimates in the hot season than that in the cool season. That may be attributed to the constituents of the complex mix of PM_2.5_ and PM_10_, which may vary by season. The exact PM_2.5_ compositional difference in different seasons is currently under investigation.

The varying magnitude of misclassifications of clinical diagnosis and ICD-10 codes may have introduced bias. To mitigate this bias, one ICD-10 code rematch and validate mechanism was introduced in our research. While these diagnoses made by physicians may not be accurate and completely consistent. Further studies are needed to validate these diagnoses.

Our research had limitations. First, we collected only three years’ of data for the association analysis between air pollution and children respiratory outpatient visits; the GAM model, therefore, may have some instability [33]. Second, as in many previous time-series studies, we used available ambient monitoring data to assess the children’s exposure to air pollutants. As a result, several issues may have arisen, given that ambient monitoring results differ from a child’s exposure level to air pollutants [33, 34]. Data for the assessment of weather conditions was retrieved entirely from one monitoring station. Measurement error may have substantial implications for interpreting epidemiological studies on air pollution. Third, we evaluated the association of five air pollutants with seven different hospital outpatient outcomes. In addition, moderate-to-high correlation between PM pollution and gaseous pollutants in a selected city limited our ability to separate the independent effect for each pollutant.

## Conclusions

Our findings suggest that ambient air pollutants were associated with child respiratory outpatient visits, especially for pneumonia (ICD-10 code J18), other acute lower respiratory infections (ICD-10 code J20-J21), and other diseases of upper respiratory tract (ICD-10 code J30-J39). Furthermore, our results suggest that the effect estimates in the transition season were stronger than in cold or hot seasons, and that the estimates increase with age in children. To protect the health of children, local authorities should take more measures to control air pollutant emissions.

## Abbreviations

df: degrees of freedom
GAM: generalized additive model
ICD-10: International Classification of Diseases, 10th Revision
NO_2_: nitrogen dioxide
O_3_: 0zone
PM: particulate matter
PM_10_: inhalable particles
PM_2.5_: fine particles
SO_2_: sulfur dioxide
